# Generalized Social Dilemmas: The Evolution of Cooperation in Populations with Variable Group Size

**DOI:** 10.1007/s11538-018-00545-1

**Published:** 2018-12-17

**Authors:** Mark Broom, Karan Pattni, Jan Rychtář

**Affiliations:** 1 Department of Mathematics, City, University of London, Northampton Square, London, EC1V 0HB UK; 2 Department of Mathematical Sciences, The University of Liverpool, Mathematical Sciences Building, Liverpool, L69 7ZL UK; 3 Department of Mathematics and Statistics, The University of North Carolina at Greensboro, Greensboro, NC 27412 USA

**Keywords:** Evolutionary game, Cooperation, Public goods game, Prisoner’s Dilemma, Hawk–Dove game, 91A22, 92D15

## Abstract

Evolutionary game theory is an important tool to model animal and human behaviour. A key class of games is the social dilemmas, where cooperation benefits the group but defection benefits the individual within any group. Previous works have considered which games qualify as social dilemmas, and different categories of dilemmas, but have generally concentrated on fixed sizes of interacting groups. In this paper, we develop a systematic investigation of social dilemmas on all group sizes. This allows for a richer definition of social dilemmas. For example, while increasing a group size to include another defector is always bad for all existing group members, extra cooperators can be good or bad, depending upon the particular dilemma and group size. We consider a number of commonly used social dilemmas in this context and in particular show the effect of variability in group sizes for the example of a population comprising negative binomially distributed group sizes. The most striking effect is that increasing the variability in group sizes for non-threshold public goods games is favourable for the evolution of cooperation. The situation for threshold public goods games and commons dilemmas is more complex.

## Introduction

Evolutionary game theory has proved to be a valuable way of modelling behaviour with both animal and human populations (Maynard Smith 1982; Maynard Smith and Price 1973). A particular problem that has attracted widespread attention is the evolution of altruistic or cooperative behaviour (Trivers 1971; Axelrod 2006; Nowak 2006a). An important class of evolutionary game has been developed to address these issues, namely the social dilemmas (Kerr et al. 2004). Here groups of individuals meet with two available strategies, which can be termed Cooperate and Defect. Cooperative behaviour benefits the group, at some cost to the individual. The group therefore as a whole faces a dilemma because, collectively, unselfish behaviour, i.e. cooperating, would benefit the entire group but, individually, selfish behaviour, i.e. defecting, would leave the individual better off.

In a social dilemma, there are several different interpretations of cooperative behaviour (Kerr et al. 2004). The one considered in Pena et al. (2016) is a slight variation to the *individual-centred* interpretation of Kerr et al. (2004) where the effect of cooperation is measured through the change in fitness of individuals rather than the group. These papers consider social dilemmas and their properties in detail for a fixed group size; for instance, what is the effect of a change of strategy of a single individual, to the individual and to others in the group? In a social dilemma, an individual interacting in a group is usually better off being selfish, irrespective of the behaviour of the other individuals. We note that there are exceptions, for instance, in the Volunteer’s Dilemma or “threshold” games, where a minimum number of cooperators is required to generate a resource (we see examples of this in Sect. 3).

There are many examples of this kind of conflict between the group and individual in biology. For example, in cooperative hunting, prey is equally shared between the hunters regardless of the effort they put in Packer et al. (1990), Stander (1992), Creel (1997) and Bednarz (1988) and, therefore, the hunters are tempted to put in less effort. Groups form to fight over food, e.g. in African wild dogs (Ginsberg and Macdonald 1990) or roadrunners (Kelley et al. 2011). Within human societies, where the most complex interactions take place, examples include the exploitation of natural resources (Kollock 1998) and hunting and the sharing of food in hunter–gatherer societies (Boehm and Boehm 2009). Such general multi-player games have been studied recently (see for example Gokhale and Traulsen (2010), Gokhale and Traulsen (2014) and Broom and Rychtář (2013), Chapter 9).

In this paper, we extend the interpretation given in Pena et al. (2016) to a population where the size of the group is variable. This is clearly a feature of real populations, such as those described above. It also allows for a more general description of social dilemmas; for example, what is the effect of adding or removing an individual (either a defector or a cooperator) to a group? What is the effect of different group size distributions within a population on the evolution of cooperative behaviour? We consider various examples of social dilemmas that use this interpretation of cooperation, as well as a particular population where group size follows a negative binomial distribution.

## What is a Social Dilemma?

The social dilemmas that we consider in this paper involve two strategies called Cooperate (*C*) and Defect (*D*). In Pena et al. (2016), the authors consider a group of individuals of fixed size *d*, which is some combination of cooperators and defectors. In their work, they outline conditions on this game to be a social dilemma, as we describe below, and we will build upon their definitions for the case of variable group size. The games considered are *symmetric* (Broom et al. 1997), which means that only an individual’s own strategy and the combination of the strategies used by the other players matters, i.e. there is no relevance to any ordering of the other players. In particular, the payoff to a cooperator (defector) in a group with *c* other cooperators and *d* other defectors is written as $$R_\mathrm{C}(c,d)$$$$(R_\mathrm{D}(c,d))$$. When the type of the focal individual does not need to be specified, $$R_*(c,d)$$ will be used instead.

The Cooperate and Defect strategies depend upon the exact interpretation of cooperation used, which imposes conditions on the payoffs received by a focal individual. In particular, the conditions restrict how the payoffs to a focal individual should change when the group it is present in changes. We consider the change in payoff for three different scenarios: the composition of the group changes but its size remains the same; the number of defectors in the group changes; and the number of cooperators in the group changes. Below we describe various conditions for a game to be considered a social dilemma.

### Conditions for Groups of Fixed Size

For groups of fixed size where the composition of the group changes, the conditions that specify the change in payoff to a focal individual are given by Pena et al. (2016) and are based on the premise that the focal individual, regardless of its own strategy, prefers group members who cooperate. For groups of size $m+1$, this condition is given by

1 $$\begin{aligned} R_*(c,m-c) \le R_*(c',m-c')\quad \text {for } 0\le c < c' \le m, \end{aligned}$$

which states that the payoff to a focal individual may not decrease if defectors are replaced by cooperators in a group of fixed size. To ensure that cooperation has a chance to evolve, the following additional condition is imposed by Pena et al. (2016)

2 $$\begin{aligned} R_\mathrm{C}(m,0) > R_\mathrm{D}(0,m). \end{aligned}$$

This condition says that a cooperator in a group of cooperators has a strictly higher payoff than a defector in a group of defectors, i.e. the best possible situation for cooperators yields a higher reward than the worst possible situation for defectors. Conditions (1) and (2) combined give a social dilemma in Pena et al. (2016). An equivalent way of expressing condition (1) without *m* and, therefore, more suitable when talking about groups of variable size is the following

3 $$\begin{aligned} R_*(c,d) \le R_*(c+1,d-1) \quad c\ge 0, \quad d >0. \end{aligned}$$

Note here that the group size remains the same because adding a cooperator is compensated for by removing a defector.

### Conditions for Changing the Number of Defectors

In the context of variable group sizes, the conditions for cooperation when the number of defectors changes has not been specified before and will therefore be specified here as follows. We assume that defectors are completely parasitic, and so bring additional costs but no additional benefits. Thus there is no circumstance when an additional defector will enhance the payoff to any member of the group. This can be written as

4 $$\begin{aligned} R_*(c,d+1) \le R_*(c,d) \quad c,d\ge 0. \end{aligned}$$

Here while defectors bring only additional costs, cooperators may bring both additional costs and benefits. We note that it is possible to envisage a situation where there is a continuum of strategies with both cooperative and defective elements, and so whether an individual is a defector or not is relative to the behaviour of others. This is not the type of situation we wish to investigate here; hence, the condition used clearly separates defectors from cooperators, where defectors are an unambiguously harmful presence in a group.

### Conditions for Having a Social Dilemma

Conditions (3) and (4) could be understood as definitions of what constitutes strategies of cooperation and defection in multi-player games. In order for the dilemma to be present, there must also be some temptation to defect. According to (Nowak 2012), the temptation exists if either one of the following is true

5 $$\begin{aligned} R_\mathrm{D}(c, d-1)&> R_\mathrm{C}(c-1,d), \end{aligned}$$

6 $$\begin{aligned} R_\mathrm{D}(c,d)&>R_\mathrm{C}(c,d). \end{aligned}$$

Condition (5) means that, in a given group, defectors do better than cooperators. Condition (6) means that, in a given group, switching from cooperator to defector improves the payoff of the switching individual.

Following Nowak (2012), we shall call a game a social dilemma if (5) holds for all $c>0, d>0$ and (6) holds for all $c+d > 0$. If conditions (6) and (5) hold only for some values of *c*, *d*, we will call the game a relaxed social dilemma. When describing each game in Sect. 3, we shall indicate conditions when the game is a social dilemma for all group sizes above 1.

### Conditions for Changing the Number of Cooperators: Two Types of Social Dilemma

As discussed above, the conditions required for cooperation when the number of cooperators changes are less restrictive. Thus, for the interpretation of cooperation used here, a focal individual may or may not prefer adding a cooperator to the group. This interpretation of cooperation is more general and allows many more cooperative strategies to be considered. We shall divide the social dilemmas into two types: commons dilemmas and public goods dilemmas, which we discuss in more detail in Sect. 3. We shall say that a game is a commons dilemma if and only if the addition of an extra cooperator is never beneficial to any member of the group, i.e.

7 $$\begin{aligned} R_*(c+1,d)\le R_*(c,d) \quad c,d\ge 0. \end{aligned}$$

Otherwise we shall call a game a public goods dilemma. A further distinction is possible, although we do not consider this in any detail in the cases we consider. We denote games where an additional cooperator is never harmful, i.e. where

8 $$\begin{aligned} R_*(c+1,d)\ge R_*(c,d) \quad c,d\ge 0 \end{aligned}$$

as a pure public goods dilemma, with other games being labelled as partial public goods dilemmas. For example, suppose that cooperators provide a shared resource such that adding another cooperator increases the shared resource. If the amount provided by each additional cooperator diminishes, a point will be reached where adding another cooperator results in a fall in the share that each individual receives. In this case, cooperators provide a benefit to the focal individual up to a certain point. We consider such a situation later, in Sect. 3. If cooperators always benefited the focal individual, this kind of behaviour would be excluded. Therefore, conditions given by inequalities (3) and (4) are all that is required for our interpretation of cooperation.

### Adding or Removing an Individual from a Group

By combining the conditions given by inequalities (3) and (4), the payoffs for the four possible single individual changes, i.e. where there is one more cooperator $(c+1,d)$, one more defector $(c,d+1)$, one less cooperator $(c-1,d)$ and one less defector $(c,d-1)$, can be ranked with respect to the group (*c*, *d*) for a focal individual, regardless of its type. All the possible payoff rankings are given in Table 1. For arbitrary games, there are $5!=120$ orderings; as we see in Table 1, for our games there are just 11. Different orderings or combinations of orderings can then occur for different social dilemmas, and so consideration of such orderings helps us to distinguish between them, as we shall see later.

**Table 1:** Possible payoff rankings for a social dilemma. Here we consider the payoff to individuals (either cooperator or defector) within a group of given size, compared to all four possible changes of a single individual (adding or removing a cooperator or a defector). The 11 orderings listed are all of those allowed following inequalities (3) and (4)

Ordering	$R_*(\ldots )$	$\le R_*(\ldots )$	$\le R_*(\ldots )$	$\le R_*(\ldots )$	$\le R_*(\ldots )$
1.	$c,d+1$	*c*, *d*	$c-1,d$	$c,d-1$	$c+1,d$
2.	$c,d+1$	*c*, *d*	$c-1,d$	$c+1,d$	$c,d-1$
3.	$c-1,d$	$c,d+1$	*c*, *d*	$c,d-1$	$c+1,d$
4.	$c,d+1$	$c-1,d$	*c*, *d*	$c,d-1$	$c+1,d$
5.	$c-1,d$	$c,d+1$	*c*, *d*	$c+1,d$	$c,d-1$
6.	$c,d+1$	$c-1,d$	*c*, *d*	$c+1,d$	$c,d-1$
7.	$c,d+1$	$c-1,d$	$c+1,d$	*c*, *d*	$c,d-1$
8.	$c-1,d$	$c,d+1$	$c+1,d$	*c*, *d*	$c,d-1$
9.	$c,d+1$	*c*, *d*	$c+1,d$	$c-1,d$	$c,d-1$
10.	$c,d+1$	$c+1,d$	*c*, *d*	$c-1,d$	$c,d-1$
11.	$c,d+1$	$c+1,d$	$c-1,d$	*c*, *d*	$c,d-1$

## Types of Social Dilemmas

There are two broad categories of social dilemmas that were identified in Kollock (1998) and are described below in relation to the conditions identified above.

### Public Goods Dilemmas

This dilemma involves the *production* of a public good that can be enjoyed by all group members whether or not they have contributed towards its production. This means that public goods are *non-excludable* and, in additions to this, they may also be *non-rivalrous*, whereby its consumption by one individual does not diminish its availability to another individual. A *pure* public good is both entirely non-excludable and non-rivalrous; however, in general public goods have a varying degree of both non-excludability and non-rivalrousness. For public goods dilemmas, cooperators are assumed to always contribute towards the production of the public good, while defectors do not.

For the public goods dilemmas considered here, the payoffs are of the form

9 $$\begin{aligned} R_\mathrm{C}(c,d)&= p_\mathrm{C}(c)\cdot u_\mathrm{C}(c,d) \cdot V - k_\mathrm{C}(c)\cdot K, \end{aligned}$$

10 $$\begin{aligned} R_\mathrm{D}(c,d)&= p_\mathrm{D}(c)\cdot u_\mathrm{D}(c,d) \cdot V, \end{aligned}$$

where $p_*(c)$ is the production function that determines how much of a public good *V* is produced when a focal individual is present with *c* other cooperators, $k_\mathrm{C}(c)$ is the cost function that determines the share of the cost *K* paid by a focal cooperator present with *c* other cooperators and $u_*(c,d)$ is the proportion of the total available public good a focal individual receives when present with *c* (*d*) other cooperators (defectors); we note that this is not simply a division of a fixed good between the group members, as in non-rivalrous games all individuals can obtain the full value. The public good value $V>0$ and cost $K>0$ are used as universal parameters for the different public goods games. Note that since a defector does not contribute to the production of a good, the defector production function will be set to $p_\mathrm{D}(c)=p_\mathrm{C}(c-1)$. Also, for the same reason, the defectors do not have a cost function.

In Kollock (1998), several different forms of production functions are identified. Letting $\varDelta p_\mathrm{C}(c)=p_\mathrm{C}(c)-p_\mathrm{C}(c-1)$, the production function can take the following forms:

(i) *Convex function* (accelerating or increasing returns to scale):
  11 $$\begin{aligned} \varDelta p_\mathrm{C}(c+1)>\varDelta p_\mathrm{C}(c)>0\ \forall c. \end{aligned}$$
(ii) *Linear function* (constant returns to scale):
  12 $$\begin{aligned} \varDelta p_\mathrm{C}(c+1)=\varDelta p_\mathrm{C}(c)>0\ \forall c. \end{aligned}$$
(iii) *Concave function* (decelerating or decreasing returns to scale):
  13 $$\begin{aligned} 0<\varDelta p_\mathrm{C}(c+1)<\varDelta p_\mathrm{C}(c)\ \forall c. \end{aligned}$$
(iv) *Step function*: In this case, no public good is produced if the number of cooperators, $c+1$ if our focal individual is a cooperator, is below some threshold *L*, for example, $p_\mathrm{C}(c)={\mathbf {1}}_{c+1\ge L }$ where
  14 $$\begin{aligned} {\mathbf {1}}_{c+1\ge L } = {\left\{ \begin{array}{ll} 1 &{} c+1 \ge L, \\ 0 &{} c+1 < L. \end{array}\right. } \end{aligned}$$

The public good sharing function $u_*(c,d)$ can be interpreted in two different ways. Firstly, it can be constant such that $u_\mathrm{C}(c,d)=u_\mathrm{D}(c,d)>0$. This implies that the public good is pure, i.e. non-excludable and non-rivalrous, and all group members get the same amount of the public good. Secondly, it is some non-constant function that represents a public good exhibiting excludability or rivalrousness (or both). The cost function $k_\mathrm{C}(c)$ can either be constant or non-constant. If constant, each cooperator pays the same cost regardless of the number of cooperators. If non-constant, the cost can either increase or decrease with the number of cooperators.

In Table 1, all the inequalities can appear within a public goods dilemma. The inequalities where $R_*(c-1,d)\le R_*(c,d) \le R_*(c+1,d)$ are consistent with what one might expect, because cooperators contribute towards the production of a public good. The inequalities where $R_*(c-1,d)\ge R_*(c,d)$ and/or $R_*(c,d)\ge R_*(c+1,d)$ may at first seem inconsistent; however, there are circumstances where they appear in public goods dilemmas particularly when the production function is decelerating. In this case, adding another cooperator may not increase the payoff to the focal individual.

**Table 2:** Summary of public goods dilemmas used in this paper. For each game, the values of the key payoff terms from Eqs. (9) and (10) are given, as functions of *c* and *d*. In each case, $P_\mathrm{D}(c)=P_\mathrm{C}(c-1)$

	$p_\mathrm{C}(c)$	$u_\mathrm{C}(c,d)$	$u_\mathrm{D}(c,d)$	$k_\mathrm{C}(c)$
Prisoner’s Dilemma	$c+1$	$\frac{1}{c+d+1}$	$\frac{1}{c+d+1}$	1
Prisoner’s Dilemma with variable production function	$\sum _{k=0}^{c} \omega ^k$	$\frac{1}{c+d+1}$	$\frac{1}{c+d+1}$	1
Stag Hunt	$(c+1){\mathbf {1}}_{c+1\ge L }$	$\frac{1}{c+d+1}$	$\frac{1}{c+d+1}$	1
Fixed Stag Hunt	${\mathbf {1}}_{c+1\ge L }$	$\frac{1}{c+d+1}$	$\frac{1}{c+d+1}$	1
Charitable Prisoner’s Dilemma	$c+1$	$${\left\{ \begin{array}{ll}\frac{c}{c+1}\frac{1}{c+d} &{} c > 0 \\ 0 &{} c = 0 \end{array}\right. }$$	$${\left\{ \begin{array}{ll}\frac{1}{c+d} &{} c > 0 \\ 0 &{} c = 0 \end{array}\right. }$$	1
Volunteer’s Dilemma	1	1	1	1
Threshold Volunteer’s Dilemma	${\mathbf {1}}_{c+1\ge L }$	1	1	1
Snowdrift	1	1	1	$\frac{1}{c+1}$
Threshold Snowdrift	${\mathbf {1}}_{c+1\ge L }$	1	1	$\frac{{\mathbf {1}}_{c+1< L }}{L} + \frac{{\mathbf {1}}_{c+1\ge L }}{c+1}$

#### Examples of Public Goods Dilemmas

The public goods dilemmas considered are summarized in Table 2. An explanation of each one is given in what follows.

*Prisoner’s Dilemma (PD)* (Hamburger 1973) In this game, the public good is non-excludable but rivalrous such that it is shared equally amongst all group members and grows linearly with the number of cooperators. However, unlike the reward, the cost is not shared by the cooperators. Each cooperator pays a cost *K*. The payoffs are given by

15 $$\begin{aligned} R_\mathrm{C}(c,d)&= \frac{c+1}{c+d+1}V - K, \end{aligned}$$

16 $$\begin{aligned} R_\mathrm{D}(c,d)&= \frac{c}{c+d+1}V. \end{aligned}$$

Condition (5) holds for all $c>0, d>0$ and condition (6) holds for $c+d > 0$ whenever $V/(c+d+1)<K$. Thus, whenever $V/2<K$, the Prisoner’s Dilemma is a social dilemma. Following condition (8), PD is always a pure public goods dilemma.

**Fig. 1:**
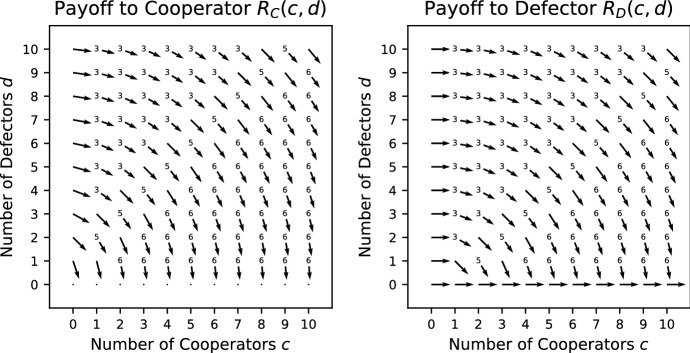
Vector field for the Prisoner’s Dilemma where $V=5/2,\ K=4/3$. The construction of the vector fields is explained in Appendix A. The direction of each vector indicates the change in group composition required to increase the payoff to a focal individual. For example, a vector pointing diagonally right indicates that an increase in payoff can be achieved by adding a cooperator or removing a defector. If the vector has more rightward bias, adding a cooperator has a bigger effect than removing a defector, and, if the vector has more downward bias, the opposite is true. Where a number is added at the base of an arrow, it indicates the specific ordering of the payoffs from Table 1. For ties, there can be more than one ordering, in which case no number is given

In Fig. 1, the vector field for the Prisoner’s Dilemma is shown. The way in which the vector field is constructed is explained in Appendix A. Each vector gives the direction in which the largest increase in payoff to the focal individual can be achieved. Given that the *x*-axis (*y*-axis) indicates the number of cooperators (defectors) present with the focal individual, a vector pointing to the right (left) indicates that the payoff can be increased by adding (removing) a cooperator and a vector pointing downwards indicates that an increase in payoff can be achieved by removing a defector. Note that there are no vectors pointing upwards as adding a defector cannot increase the payoff. A vector pointing diagonally right (left) and downwards indicates that a payoff can be increased by either adding (removing) a cooperator and removing a defector. However, if it is biased more to the right (left) than downwards, adding (removing) a cooperator has a bigger effect than removing a defector.

For any game, we can find which payoff rankings in Table 1 appear by making pairwise comparisons between the different positions (we explain this in more detail in a small number of cases for illustration). In this game, removing a cooperator or adding a defector can never improve the payoff, whereas adding a cooperator or removing a defectors can never make it worse (see the vector fields of Fig. 1 where all vectors are pointing down, right or diagonally right and down). Thus every situation satisfies one of the four orderings 3–6. Careful consideration shows that adding a cooperator cannot be better than removing a defector simultaneously with removing a cooperator being better than adding a defector. This removes case 4, so only cases 3, 5 and 6 are possible (which can all occur, as we see in Fig. 1).

*Prisoner’s Dilemma with variable production function (PDV)* (Archetti and Scheuring 2012) This is similar to the Prisoner’s Dilemma, but the public good can grow at a varying rate with respect to the number of cooperators. The payoffs are given by

17 $$\begin{aligned} R_\mathrm{C}(c,d)&= -K + \frac{V}{c+d + 1}\sum _{n=0}^{c}\omega ^{n}, \quad \omega >0, \end{aligned}$$

18 $$\begin{aligned} R_\mathrm{D}(c,d)&= \frac{V}{c+d + 1}\sum _{n=0}^{c-1} \omega ^{n}, \quad \omega > 0. \end{aligned}$$

The production function is convex for $\omega > 1$, concave for $\omega <1$ and linear for $\omega =1$ (this gives the original Prisoner’s Dilemma).

Condition (5) holds for all $c>0, d>0$ and condition (6) holds for $c+d > 0$ whenever $\frac{V}{c+d+1}\omega ^c<K$. Thus, when $\omega \le 1$ and $V/2<K$, PVD is a social dilemma. When $\omega >1$ and $\frac{V}{2}\omega ^N<K$, PVD is a social dilemma in populations where the maximum interacting group size is *N*, if such a maximum exists. For $w \ge 1$, PVD is a pure public goods dilemma.

**Fig. 2:**
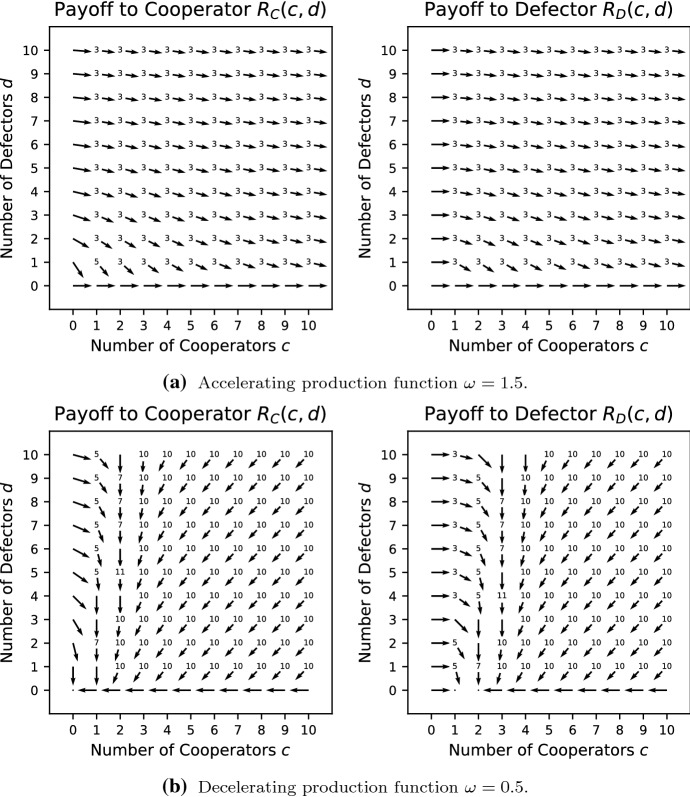
Vector field for the Prisoner’s Dilemma with variable production function where $V=5/2,\ K=4/3$

Consider the case where $\omega =0.5$ for this game whose vector field is shown in Fig. 2b. Here, more payoff rankings in Table 1 appear because the payoff can be increased by either adding or removing a cooperator, in addition to removing a defector. In particular, this is evident by both left and right pointing vectors in the vector fields. The payoff function is concave, which means that it is not possible for adding a cooperator and removing a cooperator to both be beneficial; this excludes cases 1, 2 and 9. In addition calculations show that if adding a cooperator is better than losing a defector then adding a defector is better than losing a cooperator, which excludes case 4 (as well as the already excluded case 1), and if adding a cooperator improves the payoff, then adding a defector is better that removing a cooperator, which excludes case 6. Case 8 is difficult to achieve; it requires adding a cooperator and a defector to be beneficial at $(c-1,d)$ and adding a cooperator to be harmful at (*c*, *d*) and is only achievable on the boundary between other regions; it does not occur in this case. Thus we have cases 3, 5, 7, 10 and 11 remaining, which have all been shown numerically to be possible (see Fig. 2b).

*Stag Hunt (SH)* (Pacheco et al. 2009) This is a Prisoner’s Dilemma where the production function is a step function such that at least $L>1$ cooperators are required for the public good to be produced. The cooperators always pay a cost *K* whether the threshold is met or not. The payoffs are given by

19 $$\begin{aligned} R_\mathrm{C}(c,d)&= {\left\{ \begin{array}{ll} \frac{c+1}{c+d+1}V - K &{}\quad c+1 \ge L, \\ -K &{}\quad c+1 < L; \end{array}\right. } \end{aligned}$$

20 $$\begin{aligned} R_\mathrm{D}(c,d) =&{\left\{ \begin{array}{ll} \frac{c}{c+d+1}V &{}\quad c \ge L, \\ 0 &{}\quad c < L. \end{array}\right. } \end{aligned}$$

**Fig. 3:**
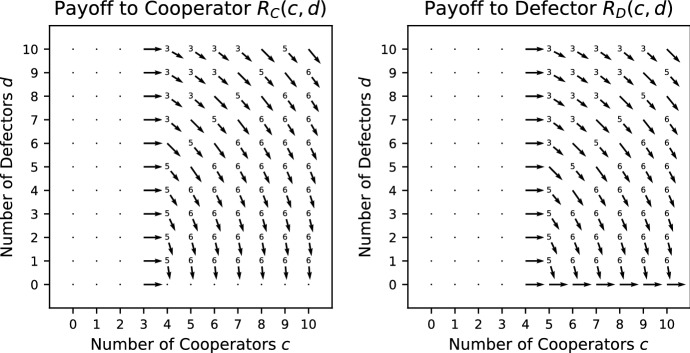
Vector field for the Stag Hunt with threshold ${L} = 5$ where $V = 5/2$; $K = 4/3$

Condition (5) holds for all $c>0, d>0$. When $c<L-1$, condition (6) holds for all $d \ge 0$. When $c>L-1$ , condition (6) holds for all $d \ge 0$ whenever $V/(c+d+1)<K$, in particular whenever $V/2<K$. However, when $c=L-1$ condition (6) holds only when $K>\frac{L}{L+d}V$. Consequently, we would need $K>V$ to be true for SH to be a social dilemma, which would make it impossible for cooperation to ever evolve, following Sect. 2.1. However, as long as $V/2<K$, SH is a relaxed social dilemma. SH is always a pure public goods dilemma (Fig. 3).

*Fixed Stag Hunt (FSH)* (Pacheco et al. 2009) This is similar to the Stag Hunt but the public good is of a fixed size, i.e. it does not grow with the number of cooperators. The payoffs are given by

21 $$\begin{aligned} R_\mathrm{C}(c,d)&= {\left\{ \begin{array}{ll} \frac{V}{c+d+1} - K &{}\quad c+1 \ge L, \\ -K &{}\quad c+1 < L; \end{array}\right. } \end{aligned}$$

22 $$\begin{aligned} R_\mathrm{D}(c,d) =&{\left\{ \begin{array}{ll} \frac{V}{c+d+1} &{}\quad c \ge L, \\ 0 &{}\quad c < L. \end{array}\right. } \end{aligned}$$

**Fig. 4:**
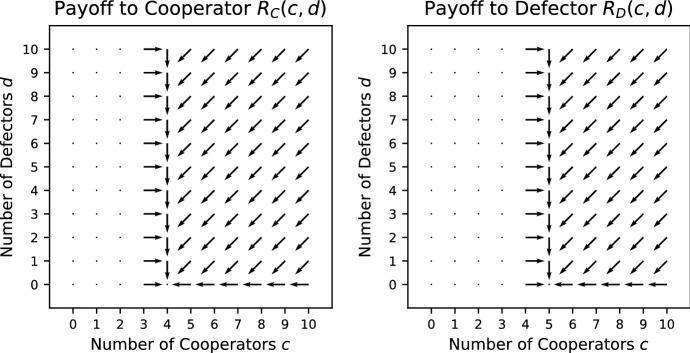
Vector field for the Fixed Stag Hunt with threshold $L = 5$ where $V = 5/2$; $K = 4/3$

Condition (5) holds for all $c>0, d>0$. When $c<L-1$ or $c>L-1$, condition (6) holds for all $d \ge 0$. When $c=L-1$, condition (6) holds only when $K>\frac{1}{L+d}V$. Consequently, as long as $V/2<K$, FSH is a social dilemma. FSH is never a pure public goods dilemma. See Fig. 4 for the vector field of FSH.

*Charitable Prisoner’s Dilemma (CPD)* (Broom et al. 2015) This is an extension of the Prisoner’s Dilemma where the public good is now excludable so that a cooperator cannot consume its own contribution to the public good. In other words, the cooperators behave charitably by giving away their contribution to the other members of the group. Furthermore, it is assumed that a cooperator will still pay the cost *K* when alone but not receive the public good. The payoffs are then given by

23 $$\begin{aligned} R_\mathrm{C}(c,d)&= {\left\{ \begin{array}{ll} \frac{c}{c+d}V - K &{}\quad c > 0, \\ -K &{}\quad c =0; \end{array}\right. } \end{aligned}$$

24 $$\begin{aligned} R_\mathrm{D}(c,d) =&{\left\{ \begin{array}{ll} \frac{c}{c+d}V &{}\quad c > 0, \\ 0 &{}\quad c = 0. \end{array}\right. } \end{aligned}$$

**Fig. 5:**
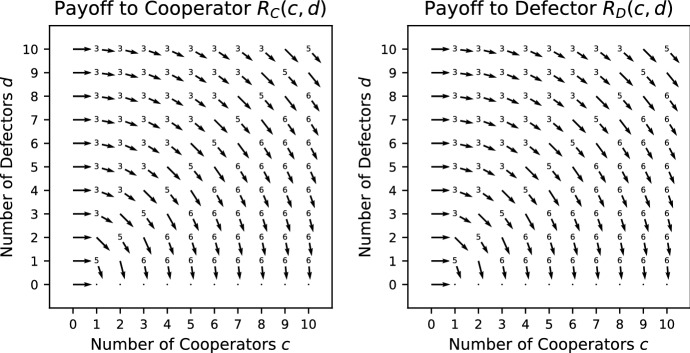
Vector field for Charitable Prisoner’s Dilemma where $V = 5/2$; $K = 4/3$

Condition (5) holds for all $c>0, d>0$ and condition (6) holds for all $c+d > 0$. Thus, CPD is a social dilemma. CPD is also always a pure public goods dilemma. See Fig. 5 for the vector field of CPD.

*Volunteer’s Dilemma (VD)* (Diekmann 1985) Here the public good is pure and of fixed size; it is provided if and only if there is at least one cooperator in the group to pay the cost *K*, which all cooperators do. The payoffs are given by

25 $$\begin{aligned} R_\mathrm{C}(c,d)&=\ V-K, \end{aligned}$$

26 $$\begin{aligned} R_\mathrm{D}(c,d) =&{\left\{ \begin{array}{ll} V &{}\quad c > 0, \\ 0 &{}\quad c = 0. \end{array}\right. } \end{aligned}$$

**Fig. 6:**
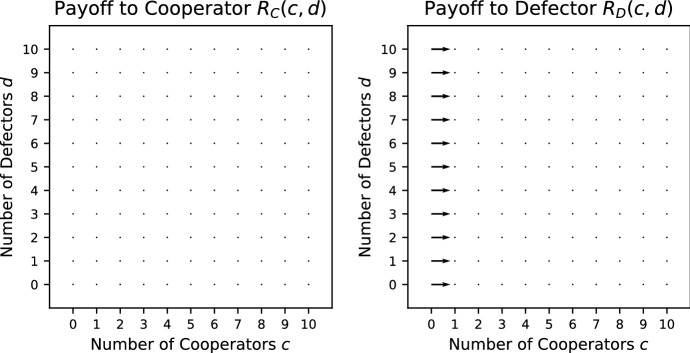
Vector field for the Volunteer’s Dilemma where $V = 5/2$; $K = 4/3$

Condition (5) holds for all $c>0, d>0$. When $c>0$, condition (6) holds for all $d \ge 0$. When $c=0$, condition (6) holds only when $K>V$. Consequently, VD is a relaxed social dilemma. VD is always a pure public goods dilemma. See Fig. 6 for the vector field of VD.

*Threshold Volunteer’s Dilemma (TVD)* (Archetti and Scheuring 2012) This is a Volunteer’s Dilemma with a threshold production function such that $L>1$ cooperators are required to provide the public good. The cooperators always pay cost *K* regardless of whether the threshold is met or not. The payoffs are given by

27 $$\begin{aligned} R_\mathrm{C}(c,d)&= {\left\{ \begin{array}{ll} V - K &{}\quad c+1 \ge L, \\ -K &{}\quad c+1 < L; \end{array}\right. } \end{aligned}$$

28 $$\begin{aligned} R_\mathrm{D}(c,d)&= {\left\{ \begin{array}{ll} V &{}\quad c \ge L, \\ 0 &{}\quad c < L. \end{array}\right. } \end{aligned}$$

**Fig. 7:**
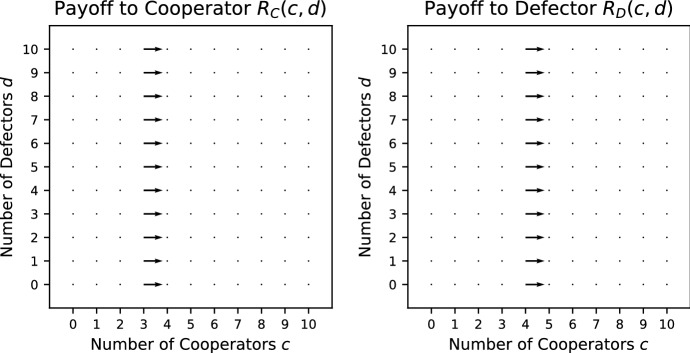
Vector field for the Threshold Volunteer’s Dilemma with $L = 5$ where $V = 5/2$; $K = 4/3$

Condition (5) holds for all $c>0, d>0$. When $c\ne L-1$, condition (6) holds for all $d \ge 0$. When $c=L-1$, condition (6) holds only when $K>V$. Consequently, TVD is a relaxed social dilemma. TVD is always a pure public goods dilemma. See Fig. 7 for the vector field of TVD.

*Snowdrift (S)* (Archetti and Scheuring 2012) This game is a Volunteer’s Dilemma where the cost is shared equally between all cooperators in the group. The payoffs are given by

29 $$\begin{aligned} R_\mathrm{C}(c,d)&= V - \frac{K}{c+1}, \end{aligned}$$

30 $$\begin{aligned} R_\mathrm{D}(c,d)&= {\left\{ \begin{array}{ll}V &{} c > 0, \\ 0 &{} c = 0. \end{array}\right. } \end{aligned}$$

**Fig. 8:**
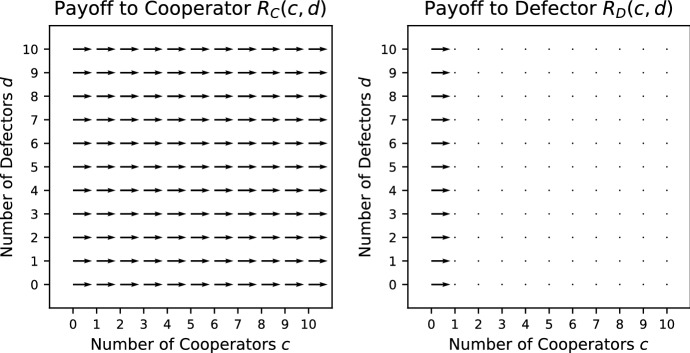
Vector field for the Snowdrift game where $V = 5/2$; $K = 4/3$

Condition (5) holds for all $c>0, d>0$. When $c>0$, condition (6) holds for all $d \ge 0$. When $c=0$, condition (6) holds only when $K>V$. Consequently, S is a relaxed social dilemma. S is always a pure public goods dilemma. See Fig. 8 for the vector field of S.

*Threshold Snowdrift (TS)* (Souza et al. 2009) This is a Snowdrift game with a threshold production function such that at least $L>1$ cooperators are required to produce the public good. The cost is shared equally between the cooperators if the threshold is met; otherwise, each cooperator pays *K* / *L*. The payoffs are given by

31 $$\begin{aligned} R_\mathrm{C}(c,d)&= {\left\{ \begin{array}{ll} V - \frac{K}{c+1} &{}\quad c+1 \ge L, \\ -\frac{K}{L} &{}\quad c+1 < L; \end{array}\right. } \end{aligned}$$

32 $$\begin{aligned} R_\mathrm{D}(c,d)&= {\left\{ \begin{array}{ll} V &{}\quad c \ge L, \\ 0 &{}\quad c < L. \end{array}\right. } \end{aligned}$$

**Fig. 9:**
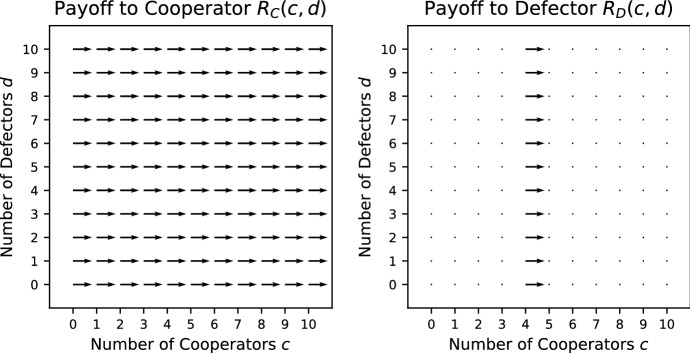
Vector field for the Threshold Snowdrift game with threshold $L = 5$ where $V = 5/2$; $K = 4/3$

Condition (5) holds for all $c>0, d>0$. When $c\ne L-1$, condition (6) holds for all $d \ge 0$. When $c=L-1$, condition (6) holds only when $K>V/L$. Consequently, TS is a social dilemma when $K>V/L$. TS is always a pure public goods dilemma. See Fig. 9 for the vector field of TS.

### Commons Dilemmas

Commons dilemmas were popularized by Hardin (1968), who gave an example of herders with access to a common parcel of land. Each herder is interested in putting as many of their cows on that land as possible because they receive the benefit of each additional cow, but the damage to the commons is shared by the group. However, if all the herders choose this option, the commons will be damaged irreparably and all will suffer. In an evolutionary biology context, consider the example of parasitic viruses residing in a bacterial cell host (Dionisio and Gordo 2006). A virus can be more competitive and use up more resources resulting in a larger number of direct progeny. If all the viruses did this, the host will die faster and the total number of progeny will be smaller. On the other hand, the viruses can be less competitive and use less resources. In this case, the number of direct progeny would be smaller, but the total number of progeny would be larger. These examples are representative of commons dilemmas in general; in particular, they involve the use of commons that are resources freely available for any group member to consume. However, commons are *rivalrous*; therefore, its consumption by one individual diminishes its availability for another individual. The dilemma here is that the group is better off if a common is used in an equitable manner, but the individual is better off being greedy and having the entire common to itself.

For commons dilemmas, it will be assumed that cooperators consume a common resource in an equitable manner and defectors do not. In Table 1, the inequalities where $R_*(c+1,d)\le R_*(c,d) \le R_*(c-1,d)$ are the only ones that appear in commons dilemmas. This is because the focal individual is better off having a common to itself and therefore prefers removing a cooperator to adding a cooperator. The following is an example of a commons dilemma.

*Hawk–Dove (HD)* (Broom and Rychtář 2012) A group of cooperators (Dove) share a common resource *V* equally; however, they flee if there is a defector (Hawk) present, getting no share of the resource. A defector chases away cooperators and fights other defectors for the common resource. Each defector has an equal chance of winning the entire resource *V*, with the losers incurring a cost *K*. In this example, an additional individual of either type is an extra drain on the common resource, but a defector has a greater negative impact than a cooperator. This is thus an extreme case of our interpretation of cooperation, and the two types could perhaps also be regarded as two types of defection but causing different levels of damage. The truly cooperative act here, if it were allowed, would be for an individual to leave the group, thus increasing the fitness of the others. With the usual choice of two strategies available, however, Dove is clearly the more cooperative, and this is how we interpret it here. The payoffs are given by

33 $$\begin{aligned} R_\mathrm{C}(c,d)&= {\left\{ \begin{array}{ll} \frac{V}{c+1} &{}\quad d = 0, \\ 0 &{}\quad d > 0; \end{array}\right. } \end{aligned}$$

34 $$\begin{aligned} R_\mathrm{D}(c,d) =&\frac{V-dK}{d+1}. \end{aligned}$$

Conditions (5) and (6) hold for all $c>0, d>0$ as long as $\frac{V}{d}>K$. Consequently, when $\frac{V}{N}>K$ where *N* is the maximum group size, HD is a social dilemma.

**Fig. 10:**
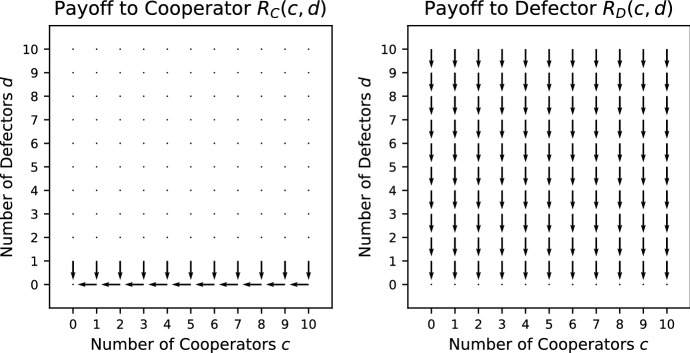
Vector field for the Hawk–Dove commons dilemma where $V = 5/2$; $K = 4/3$

In this game, the addition of an individual, cooperator or defector, can never improve the payoff, and the removal of an individual can never decrease it (see the vector field shown in Fig. 10). The addition of a cooperator is preferable to the addition of a defector, and the removal of a defector is preferable to the removal of a cooperator. This completely specifies the order, in the sense that case 10 from Table 1 always holds, although there are ties (with different orderings in different situations), as the absence of numbers by the arrows in Fig. 10 indicates. Note that there are no vectors pointing diagonally left and down in Fig. 10 because the only time it is beneficial to get rid of the cooperators is when there are no defectors in the group, i.e. $d=0$.

In the above example, and the general description of a commons dilemma in the text above, there is no production of a public good, as opposed to in the public goods dilemmas from Sect. 3.1. The addition of another player, of whatever type, never has a positive affect on the existing players, again of whatever type. In Sect. 3.1, additional defectors were never beneficial, but additional cooperators increased rewards at least in some situations for each game. We could imagine a game with both a fixed good to be shared and production by cooperators. For example, in the Prisoner’s Dilemma we could introduce a fixed reward *fV* to be shared between all players. This would lead to the following payoffs:

35 $$\begin{aligned} R'_\mathrm{C}(c,d)&= \frac{f+c+1}{c+d+1}V - K, \end{aligned}$$

36 $$\begin{aligned} R'_\mathrm{D}(c,d)&= \frac{f+c}{c+d+1}V. \end{aligned}$$

Is this game a public goods dilemma or a commons dilemma? If we use the criterion that a game is a public goods dilemma if there are circumstances where the addition of a cooperator can increase the payoff of at least one existing individual, and is a commons dilemma otherwise, then this game is a commons dilemma if and only if $f \ge N_{max}$, where $N_{max}$ is the maximum number of other individuals in the group. Note that for the negative binomial example distributions we consider in Sect. 4.2, there is no such maximum, but for alternative distributions (e.g. binomial distributions), there would be a maximum value.

## Evolution in Social Dilemmas with Variable Group Size

In this section, we shall consider a large population from which groups of various size are selected at random. A group size is selected following some distribution (we choose a negative binomial distribution as an example), and then a group of this size selected at random from the population. We shall consider an infinite population with proportion *p* of cooperators, and thus, for a given group size *n* the number of cooperators will follow a binomial distribution with parameters *n* and *p*.

### An Arbitrary Group Size Distribution

We shall consider the following random variables. Our focal individual is in a group with *N* other individuals, *A* of them are cooperators and $B=N-A$ are defectors, so that $N+1$ is the size of the group that our focal individual is in. Further denote $N^{*}$ as the size of a randomly selected group from the population. There is an important distinction between the two concepts. In Broom et al. (2015), $N+1$ was called the group size from the individual’s perspective and $N^{*}$ was called the group size from the observer’s perspective; in Peña and Nöldeke (2016), $N+1$ was referred to as the “experienced group size” and $N^{*}$ as simply the group size.

We shall further consider the *incentive function* for the strategy Cooperate, which is the level of advantage of playing Cooperate over Defect, in a population that has proportion of cooperators *x*

37 $$\begin{aligned} h_{C-D}(x)=R_{C}(x)-R_{D}(x), \end{aligned}$$

where $R_{C}(x)$ and $R_{D}(x)$ are the expected payoffs to a cooperator and a defector, respectively, in a population with proportion *x* cooperators. We shall seek evolutionarily stable strategies (ESSs) within such a population. If this incentive function is positive at $x=1$ (negative at $x=0$), then Cooperate (Defect) is a pure strategy ESS. For $0<x^{*}<1$, then $x^{*}$ is an ESS (for generic games) if and only if the incentive function takes value 0, and has negative derivative with respect to *x*, at $x=x^{*}$ [see, for example, Broom and Rychtář 2013, Chapter 7].

Thus from the different games described in Sect. 3, we have the incentive functions shown in Table 3. In each case, these are functions of *x*, since the distribution of A depends upon it.

**Table 3:** General form of the incentive function $h_{*,C-D}$ in terms of the parameters and probabilities for and expectations of random variables related to group size and composition, where $*$ is the specific game. The final column gives the evaluation of this for negative binomial incentive functions. The terms $S_{1}, S_{2}$ and $S_{3}$ are summations shown in Appendix B, $S_4=\frac{1-p}{p(1-x)}$ and $S_5 = \frac{1-p}{1-p+xp}$.

Game	General incentive function ($h_{*,C-D}$)	Incentive function for negative binomial
PD	$\displaystyle VE\left[ \frac{1}{N+1} \right] -K$	$$ {\left\{ \begin{array}{ll} -V (1-p)\ln (1-p)/p - K &{} s = 1,\\ V \frac{(1-p)}{(s-1)p} \left( 1- (1-p)^{s-1} \right) -K &{} s \ne 1. \end{array}\right. } $$
PDV	$\displaystyle VE\left[ \frac{w^{A}}{N+1} \right] -K$	$$ {\left\{ \begin{array}{ll} -V \frac{1-p}{p(wx+1-x)} \ln (1-p(wx+1-x)) - K &{} s = 1\\ V \frac{(1-p)(1- (1-p(wx+1-x))^{s-1})}{(s-1)p(wx+1-x)} \left( \frac{1-p}{1-p(wx+1-x)} \right) ^{s-1}-K &{} s \ne 1. \end{array}\right. } $$
SH	$$\displaystyle VLE\left[ \frac{1}{N+1}| A=L-1 \right] P[A=L-1]-K $$	$$ VL S_{1} -K$$
FSH	$\displaystyle VE\left[ \frac{1}{N+1}| A=L-1 \right] P[A=L-1]-K$	$V S_{1} -K$
CPD	$\displaystyle -K$	$-K$
VD	$\displaystyle VP[A=0]-K$	$V \left( \frac{1-p}{1-p+xp}\right) ^{s}-K$
TVD	$\displaystyle VP[A=L-1]-K$	$V {L+s-2 \atopwithdelims ()L-1} \left( \frac{1-p}{1-p+xp}\right) ^{s} \left( \frac{xp}{1-p+xp}\right) ^{L-1}-K$
S	$\displaystyle VP[A=0]-K E\left[ \frac{1}{A+1} \right]$	$$ {\left\{ \begin{array}{ll} V \frac{1-p}{1-p+xp} ~ - K \frac{1-p}{xp} \ln \left( \frac{1-p+xp}{1-p} \right) &{} s = 1\\ V \left( \frac{1-p}{1-p+xp}\right) ^{s} ~ - K \frac{1-p}{(s-1)xp} \left( 1-\left( \frac{1-p}{1-p+xp}\right) ^{s-1} \right) &{} s \ne 1 \end{array}\right. } $$
TS	$$\displaystyle VP[A=L-1]-$$$$\displaystyle \phantom {VV}K \left( P[A<L-1]/L+ P[A \ge L-1]E\left[ \left. \frac{1}{A+1}\right| A \ge L-1 \right] \right) $$	$$ V {L+s-2 \atopwithdelims ()L-1} \left( \frac{1-p}{1-p+xp}\right) ^{s} \left( \frac{xp}{1-p+xp}\right) ^{L-1} - K (S_{2}/L+S_{3})$$
HD	$$\displaystyle V \left( -E\left[ \frac{1}{N+1-A} \right] + P[A=N] E\left[ \frac{1}{N+1}| A=N \right] \right) + $$$$\displaystyle \phantom {vv}K \left( 1- E\left[ \frac{1}{N+1-A} \right] \right) $$	$$ {\left\{ \begin{array}{ll} V[S_4\ln (S_5) -\frac{1-p}{xp}\ln (1-xp)] + K[1+S_4\ln (S_5)] &{} s= 1\\ V\left[ -\frac{S_4(1-S_5^{s-1} )}{(s-1)} + \frac{1-p}{xp(s-1)} \left( \frac{1-p}{1-xp} \right) ^{s-1} \left( 1- (1-xp)^{s-1} \right) \right] &{} \\ \qquad + K \left[ 1-\frac{S_4(1-S_5^{s-1})}{(s-1)} \right] &{} s\ne 1 \end{array}\right. } $$

### A Negative Binomial Group Size Distribution

To proceed, we need to assume a distribution for $N^{*}$. We shall assume that $N^{*}$ follows a negative binomial distribution; in particular, suppose that $N^{*} \sim NB(s-1,p)$. Then

38 $$\begin{aligned} P[N=n] \propto (n+1)P[N^{*}=n+1], \end{aligned}$$

since clearly if there are *n* other individuals in a focal individual’s group the total group size is $n+1$, and there are $n+1$ individuals in such a group which experiences that group size (a particular individual is more likely to be selected to be part of a given large group than a given small one). Thus

39 $$\begin{aligned} P[N=n] \propto (n+1) {n+s-1 \atopwithdelims ()n+1} (1-p)^{s-1} p^{n+1} \propto {n+s-1 \atopwithdelims ()n} (1-p)^{s} p^{n} \end{aligned}$$

and so $N \sim NB(s,p)$ (noting in (39) that to identify the distribution we need only identify the terms involving *n*, as the terms independent of *n* must be such that all probabilities add to 1).

Using the above distribution, we obtain the incentive functions shown in Table 3. For details of the working, see Appendix B.

Note that the mean and variance of *N* are, respectively,

40 $$\begin{aligned} \mu _{N}=\frac{ps}{1-p},\quad \sigma ^{2}_{N}=\frac{ps}{(1-p)^{2}}. \end{aligned}$$

Thus we have

41 $$\begin{aligned} p=\frac{\mu }{\mu +s} \Rightarrow \sigma ^{2}_{N}=\frac{\mu (\mu +s)}{s}. \end{aligned}$$

Using the above negative binomial distribution, we now consider all of our games. We consider how the incentive function, and so the relative performance of cooperators to defectors, changes as we change the variability of the group size.

For a given $\mu$, we consider three different values of the parameter *s*:

i. $$s=1 \Rightarrow \sigma ^{2}_{N}=\mu (\mu +1);$$
ii. $$s=\mu \Rightarrow \sigma ^{2}_{N}=2 \mu ;$$
iii. $$s \rightarrow \infty \Rightarrow \sigma ^{2}_{N}=\mu .$$

**Fig. 11:**
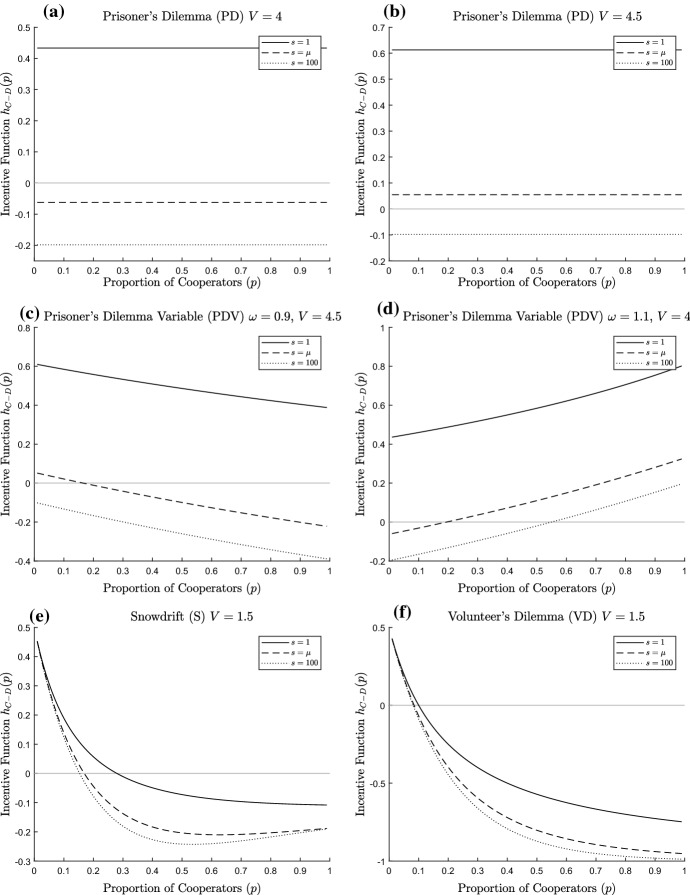
Incentive function for non-threshold public goods dilemmas. For each figure, the solid line represents $s=1$, the dashed line $s=\mu$ and the dotted line $s=100$; the light grey line is the horizontal axis, to indicate critical values of the incentive functions. **a** PD $V=4, K=1, \mu =5$, **b** PD $V=4.5, K=1, \mu =5$, **c** PDV $w=0.9, V=4.5, K=1, \mu =5$, **d** PDV $w=1.1, V=4, K=1, \mu =5$, **e** S $V=1.5, K=1, \mu =5$, **f** VD $V=1.5, K=1, \mu =5$

**Fig. 12:**
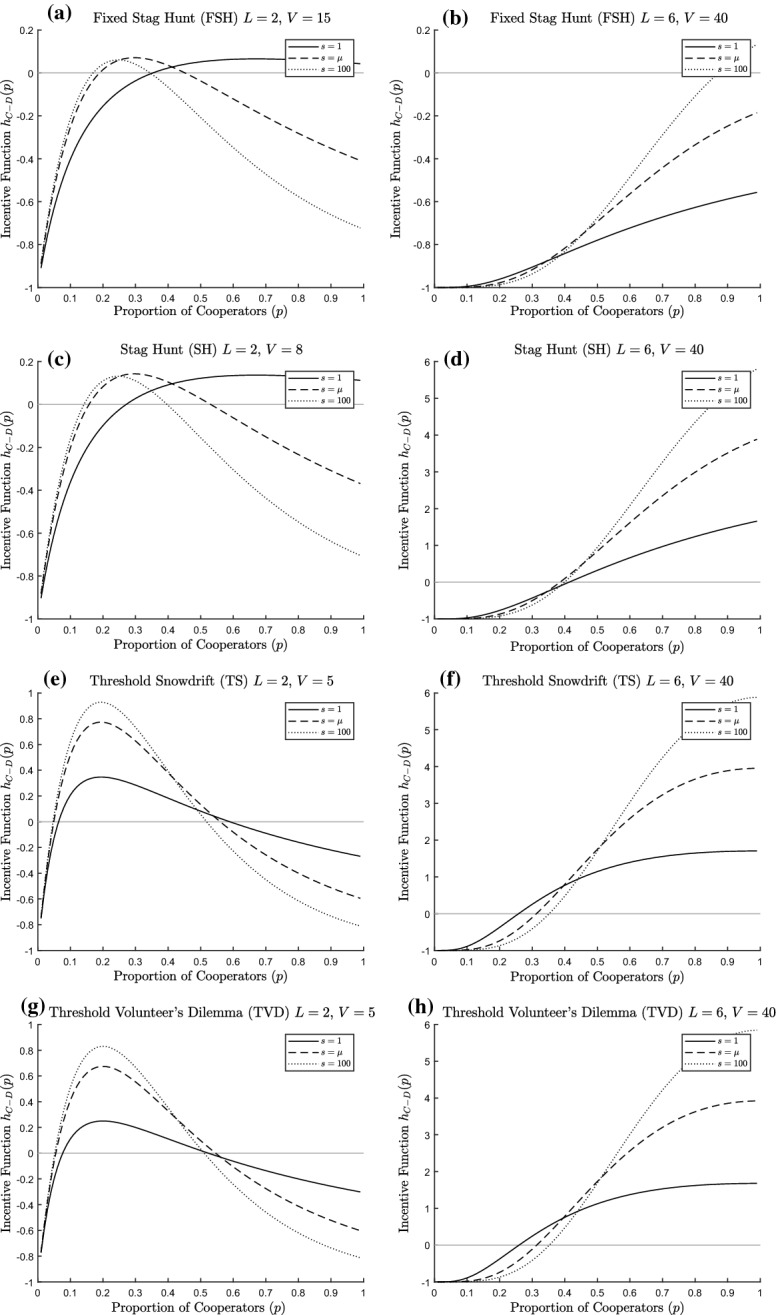
Incentive function for threshold public goods dilemmas. For each figure, the solid line represents $s=1$, the dashed line $s=\mu$ and the dotted line $s=100$; the light grey line is the horizontal axis, to indicate critical values of the incentive functions. **a** FSH $L=2, V=15, K=1, \mu =5$, **b** FSH $L=6, V=40, K=1, \mu =5$, **c** SH $L=2, V=8, K=1, \mu =5$, **d** SH $L=6, V=40, K=1, \mu =5$, **e** TS $L=2, V=5, K=1, \mu =5$, **f** TS $L=6, V=40, K=1, \mu =5$, **g** TVD $L=2, V=5, K=1, \mu =5$, **h** TVD $L=6, V=40, K=1, \mu =5$

**Fig. 13:**
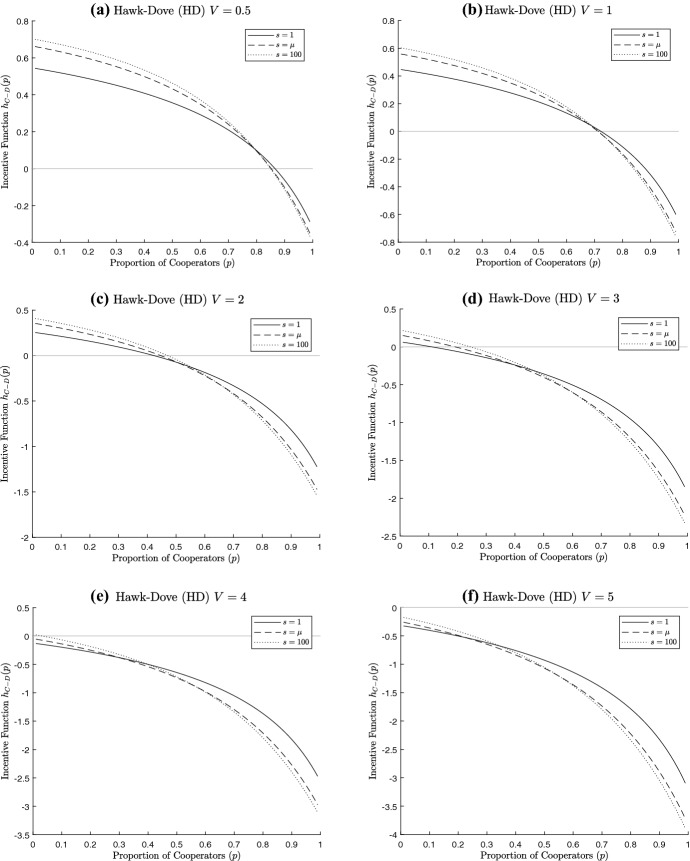
Incentive function for the Hawk–Dove game. For each figure, the solid line represents $s=1$, the dashed line $s=\mu$ and the dotted line $s=100$; the light grey line is the horizontal axis, to indicate critical values of the incentive functions. In each case, $K=1, \mu =5$ but *V* is varied. **a**$V=0.5$, **b**$V=1$, **c**$V=2$, **d**$V=3$, **e**$V=4$, **f**$V=5$

We see some example figures for all of our games in Figs. 11, 12 and 13 (note that we use $s = 100$ as our large value of s from case iii above). We try to use similar parameters where possible; however, our focus is on more interesting situations where there are mixed equilibrium values, or at least varying *s* changes the equilibria to some extent (there are many combinations of the game and parameter values which would yield defecting as the only ESS for all values of *s*, for instance). This has meant that, given the different interpretation of the parameters within the games, sometimes we have had to change the values used. We should note that this has also meant that the parameters used do not generally satisfy the conditions set out for a social dilemma for all group sizes as laid out in Sect. 2.3.

In Fig. 11, we see examples of non-threshold public goods dilemmas. In each of these figures (except for Fig. 11a, b for the PD), there is a clear trend in the incentive function (decreasing in Fig. 11c, f, decreasing then flattening/mildly increasing in Fig 11e and increasing in Fig. 11d) with the incentive functions always higher for smaller values of *s*, i.e. for more variable group sizes. We see in Fig. 11c that for the PDV with $w<1$, the incentive function is decreasing which corresponds to mixed strategy ESSs at the point where the incentive function is zero, if it exists (thus, here $s=1$ yields a pure cooperator ESS, $s=\mu$ yields a mixed ESS and $s=100$ yields pure Defect as the ESS). For $w>1$ in Fig. 11d, the incentive functions are increasing, and so a zero of the incentive function indicates a boundary between the basins of attraction of the pure ESSs that would occur under the replicator dynamics. Here for $s=1$ pure Cooperate is the unique ESS; for the other cases, there are two pure ESSs, but the boundary between the regions is lower for $s=\mu$ than for $s=100$. In the *PD*, pure Defect is the unique ESS for sufficiently large *s*; otherwise, pure Cooperate is the unique ESS.

Thus in each case, the more variable the group size, the more that cooperation is favoured. Cooperation is favoured here although in each case, the game satisfies the conditions for a social dilemma at the mean group size of 5 (except for some groups for Fig. 11d). This occurs because group variability produces enough sufficiently small groups where cooperation is actively beneficial, and this is a key reason why increased variability can favour cooperation. Note that the Charitable Prisoner’s Dilemma is an exception, as it has a simple incentive function which takes value $-K$ in all circumstances, and so here all Defect is always the unique ESS (we thus did not include a figure for this case).

In Fig. 12, we see various examples of threshold public goods dilemmas. Here the picture is more complicated. Either pure Defect is the unique ESS or there can be two ESS values, pure Defect and either a mixture of the two strategies or pure Cooperate. The effect of increased variability in group size depends upon the parameter values, in particular the relationship between the mean group size $\mu$ and the threshold *L*. If the threshold is lower than the mean group size, then the incentive function for more variable cases is initially lower than for less variable ones, and later becomes higher. Here the basin of attraction for pure Defect is larger, but the probability of cooperation in the second ESS is not monotonic in the variability of the group size. However, if *L* is higher than $\mu$ then the incentive function for more variable cases starts higher than for less variable cases, but eventually becomes lower. Here there are two pure ESSs, but no clear ordering between the boundaries between the basins of attraction (although note that we only show one figure in each case).

Figure 13 shows examples of the Hawk–Dove game, a commons dilemma. Here we see there is a unique ESS in each of the figures, with a mixed ESS in Fig. 13a–d for all three values of *s* and for one $s=100$ in Fig. 13e, and pure Defect (Hawk) in each of the other figures. As *V* increases, Defect does relatively better as we would expect. For given *V*, the incentive function for more variable cases starts lower and ends up higher than for less variable cases. The level of cooperative behaviour in the ESS does not always follow the same order, with the probability of cooperation higher for higher variability for low *V*, but lower for high *V*.

## Discussion

In this paper, social dilemmas are defined using conditions imposed on the payoff received by a focal individual. These conditions specify the relationship between the payoffs a focal individual receives when its group size and/or composition changes. In previous work in the literature, such as Kerr et al. (2004) and Pena et al. (2016), groups of fixed size are considered; here, however, variable group sizes are considered and, therefore, the conditions specified have to take changes in group size into account. We have thus developed a more general definition of social dilemmas. In particular, the conditions that the payoffs in a social dilemma need to satisfy are given in Table 1.

Two main categories of social dilemmas were then identified: public goods dilemmas and commons dilemmas. The key difference between these two dilemmas is that the former involves the production of a joint good so that the addition of cooperative players can potentially lead to benefits to existing players, and the latter does not. In public goods dilemmas, the dilemma faced by a focal individual within a group is whether or not to contribute towards the production of a public good because, regardless of what it does, it can still enjoy its benefits. However, if all individuals decide not to contribute, the entire group is worse off because there will be no public good to consume. In commons dilemmas, a common is a freely available resource that can be consumed by the entire group; however, it is rivalrous, thereby diminishing in availability as more individuals consume it. A focal individual faces the dilemma where consuming as much of the commons leaves itself better off but the group worse off. These characteristics of public goods and commons dilemmas are captured in the conditions given.

We have considered several examples of games of public goods and commons dilemmas. A bulk of the examples given are public goods games because of the diversity of the production function. In particular, multi-player public goods games have been considered in a number of papers: Hauert et al. (2002), Kurokawa and Ihara (2009), Kurokawa and Ihara (2013), Milinski et al. (2006), Santos and Pacheco (2011), Santos et al. (2008), Souza et al. (2009) and van Veelen and Nowak (2012), whereas the multi-player Hawk–Dove has only been considered in Broom et al. (2015) and Broom and Rychtář (2012). In order to visualize the behaviour found within these games, a vector field for each of the games is plotted. The vectors indicate the direction of a preferred group change for the focal individual that results in an increase in its payoff. The vector fields act as a visual substitute for the conditions given in Table 1. Some of the games are sensitive to the reward *V*, cost *K* and other parameters specific to the game, like the threshold *L*, and here the vector fields are quite effective at spotting the changes that arise; for example, see Fig. 2.

Populations with a variable group size were also considered in Peña and Nöldeke (2016). They considered a class of games involving cooperating and defecting individuals where payoffs were only a function of the number of cooperators, including two example games, one of which was the VD game as described in this paper. Their focus was on finding general results relating payoffs to group variability in the “experienced group”, equivalent to our *N*. They developed general results which show that greater variability would favour cooperation when the gain sequence (the sequence $d_{k}$ of gains from switching from defect to cooperate when there are *k* other cooperators in the group) is convex, and inhibit cooperation when it was concave. Our results are consistent with theirs for the games that are within their class, although most of our games fall outside of it.

Previously, a number of mechanisms have been identified that aid the evolution of cooperation; see, for example, Nowak (2006b). These can be divided effectively into kin selection, direct reciprocity, indirect reciprocity, population assortment and multi-level selection. None of these are at work here, so how can we explain how cooperation evolves? Firstly, we should emphasize that some of the games considered here including the Volunteer’s Dilemma and the “Prisoner’s Dilemma” allow cooperative individuals to benefit from their own contribution. Thus if this benefit is large enough, then cooperation can be favoured even in games with fixed group sizes. Perhaps the game that most reflects the original idea of the two player Prisoner’s Dilemma is the Charitable Prisoner’s Dilemma, where a cooperator cannot benefit from its own contribution. For this game, our model as considered has Defect as the unique ESS.

Thus variable group size cannot be considered as a new mechanism for the generation of cooperation. What it can do, however, is significantly enhance existing mechanisms in certain situations. In our analysis of non-threshold public goods games, the greater the variance in the group size, for fixed mean group size, either the easier it is for cooperation to dominate the population (the threshold between when Cooperate and Defect evolved as pure cooperation gave a greater basin of attraction to Cooperate) or the larger the proportion of cooperators in the ESS mixture (increasing even to one). This occurs principally because at lower group sizes it is actually beneficial for individuals to cooperate, and increased variability can make this situation more common. Thus this can help cooperation if the positive effect of smaller groups outweighs the negative effect of larger groups. One aspect of this is the effect of the increased probability of being alone, and we would argue that consideration of the payoffs to lone individuals is one aspect of a social dilemma game that it is important in realistic models to consider (though in games with fixed group sizes this is often not mentioned, as of course it never affects the results of such models). This effect of variability in group size is not unambiguous, however. There are games where increasing variability in group size can in some circumstances favour cooperation, and in other circumstances disfavour it. This happened in the Hawk–Dove game, a commons dilemma, and also threshold public goods dilemmas.

A natural extension to our work would be to consider the effect of variable group sizes in models where there is population assortment, as considered in Pena et al. (2016). This is the process by which spatial structures (see, for example, Ohtsuki et al. 2006; Nowak et al. 2010; Peña et al. 2015; Li et al. 2016) can lead to the evolution of cooperation. We would conjecture that in many cases, the addition of variability in group size would strengthen the effect of assortment in promoting cooperation.

## Constructing the Vector Fields

This section explains how the two-dimensional vector fields are constructed where the vectors indicate the direction in which an increase in payoff can be achieved. The number of defectors will be plotted on the *y*-axis and cooperators on the *x*-axis such that the following quantities

$$\begin{aligned} \delta _{*,C^+}(c,d)&= \max ( R_*(c+1,d) - R_*(c,d),0),\\ \delta _{*,C^-}(c,d)&= \max (R_*(c-1,d) - R_*(c,d),0),\\ \delta _{*,D^-}(c,d)&= \max (R_*(c,d-1) - R_*(c,d),0), \end{aligned}$$

will be used to determined the vector at some point (*c*, *d*).

In particular, if $\delta _{*,C^+}(c,d)>0$, the vector

42 $$\begin{aligned} {\mathbf {v}}_{*,C^+}(c,d)= \left( \frac{\delta _{*,C^+}(c,d)}{\delta _{*,C^+}(c,d)+\delta _{*,D^-}(c,d)}, \frac{-\delta _{*,D^-}(c,d)}{\delta _{*,C^+}(c,d)+\delta _{*,D^-}(c,d)} \right) \end{aligned}$$

is drawn, which indicates that an increase in payoff can be achieved by either adding a cooperator or removing a defector. However, adding a cooperator has a bigger effect than removing a defector if $\delta _{*,C^+}(c,d) > \delta _{*,D^-}(c,d)$.

Also, if $\delta _{*,C^-}(c,d)>0$, the vector

43 $$\begin{aligned} {\mathbf {v}}_{*,C^-}(c,d)= \left( \frac{\delta _{*,C^-}(c,d)}{\delta _{*,C^-}(c,d)+\delta _{*,D^-}(c,d)}, \frac{-\delta _{*,D^-}(c,d)}{\delta _{*,C^-}(c,d)+\delta _{*,D^-}(c,d)} \right) \end{aligned}$$

is drawn, which indicates that an increase in payoff can be achieved by either removing a cooperator or removing a defector. However, if $\delta _{*,C^+}(c,d)=\delta _{*,C^-}(c,d)=0$, neither ${\mathbf {v}}_{*,C^+}(c,d)$ nor ${\mathbf {v}}_{*,C^-}(c,d)$ are drawn and only the vector

44 $$\begin{aligned} {\mathbf {v}}_{*,D^-}(c,d)= {\left\{ \begin{array}{ll} (0,0) &{} \quad \delta _{*,D^-}(c,d) = 0,\\ (0,-1) &{} \quad \delta _{*,D^-}(c,d) \ne 0 \end{array}\right. } \end{aligned}$$

is drawn, which indicates whether removing a defector increases the payoff or not. Note that only the cases where the payoff can be increased are considered; therefore, $R_{*}(c,d+1)$ is not considered since $R_{*}(c,d+1)\le R_*(c,d)$ by Eq. (4).

## Calculations for the Negative Binomial Group Size Distribution

We first note that game *VD* is a special case of *TVD*, *PD* is a special case of *PDV* and *SH* can be obtained from *FSH* if the parameter *r* is divided by a factor *L*. For *CPD*, the incentive function is simply $-K$ in all cases, so no calculations are required. Thus we need only consider the following six cases.

*PDV:* For $s \ne 1$, we have

45 $$\begin{aligned} E \left[ \frac{w^{A}}{N+1} \right]&= \sum _{n=0}^{\infty } \sum _{y=0}^{n} \frac{w^{y}}{n+1}\frac{(n+s-1)!}{n!(s-1)!} (1-p)^{s} p^{n} \frac{n!}{y!(n-y)!}x^{y}(1-x)^{n-y} \end{aligned}$$

46 $$\begin{aligned}&=\frac{1}{s-1} \sum _{n=0}^{\infty } \frac{(n+s-1)!}{(n+1)!(s-2)!} (1-p)^{s} (p(wx+1-x))^{n} \end{aligned}$$

47 $$\begin{aligned}&=\frac{(1-p)}{(s-1)p(wx+1-x)} \left( \frac{1-p}{1-p(wx+1-x)} \right) ^{s-1} \nonumber \\&\phantom {VV}\times \sum _{m=1}^{\infty } \frac{(m+s-2)!}{(m)!(s-2)!} (1-p(wx+1-x))^{s-1} (p(wx+1-x))^{m} \end{aligned}$$

48 $$\begin{aligned}&=\frac{(1-p)}{(s-1)p(wx+1-x)} \left( \frac{1-p}{1-p(wx+1-x)} \right) ^{s-1} \times \nonumber \\&\phantom {VV}\left( 1- (1-p(wx+1-x))^{s-1} \right) . \end{aligned}$$

*PD:* In this game, $w=1$ and the above reduces to $(1-p)(1-(1-p)^{s-1})/(p(s-1))$. For $s=1$, Eq. (45) gives

49 $$\begin{aligned} E \left[ \frac{w^{A}}{N+1} \right]&=(1-p) \sum _{n=0}^{\infty } \frac{1}{n+1} (p(wx+1-x))^{n} \end{aligned}$$

50 $$\begin{aligned}&=-\frac{1-p}{p(wx+1-x)} \ln (1-p(wx+1-x)). \end{aligned}$$

For the PD game, this becomes $-(1-p)\ln (1-p)/p$.

*TVD:* To find the incentive function in this case, we need $P[A=L-1]$. Given *N*, *A* is binomially distributed with parameters *N* and *x*. Thus

51 $$\begin{aligned} P[A=y]=\sum _{n=y}^{\infty } \frac{(n+s-1)!}{n!(s-1)!} (1-p)^{s} p^{n} \frac{n!}{y!(n-y)!}x^{y}(1-x)^{n-y}. \end{aligned}$$

Setting $m=n-y$, we obtain

52 $$\begin{aligned} P[A=y]&=\sum _{m=0}^{\infty } \frac{(m+y+s-1)!}{m!(y+s-1)!} ((1-x)p)^{m} (1-(1-x)p)^{y+s} \nonumber \\&\phantom {VV}\times \frac{(y+s-1)!}{y!(s-1)!}\frac{(1-p)^{s}(xp)^{y}}{(1-(1-x)p)^{y+s}} \end{aligned}$$

53 $$\begin{aligned}&={y+s-1 \atopwithdelims ()y} \left( \frac{1-p}{1-p+xp}\right) ^{s} \left( \frac{xp}{1-p+xp}\right) ^{y}. \end{aligned}$$

Thus *A* is negatively binomially distributed with parameters *s* and $xp/(1-p+xp)$.

*S:* To find the incentive function, we need $P[A=0]$ and $E[1/(A+1)]$. From Eq. (52), we have

54 $$\begin{aligned} P[A=0]&= \left( \frac{1-p}{1-p+xp}\right) ^{s}. \end{aligned}$$

55 $$\begin{aligned} E \left[ \frac{1}{A+1} \right]&=\sum _{n=0}^{\infty } \sum _{y=0}^{n} \frac{1}{y+1}\frac{(n+s-1)!}{n!(s-1)!} (1-p)^{s} p^{n} \frac{n!}{y!(n-y)!}x^{y}(1-x)^{n-y} \end{aligned}$$

56 $$\begin{aligned}&(\text {setting } z=y+1, m=n+1) \nonumber \\&=\frac{1}{xp} \sum _{m=1}^{\infty } \sum _{z=1}^{m} \frac{(m+s-2)!}{m!(s-1)!} (1-p)^{s}p^{m} \frac{m!}{z!(m-z)!}(x)^{z}(1-x)^{m-z} \end{aligned}$$

57 $$\begin{aligned}&=\frac{1}{xp} \sum _{m=1}^{\infty } \frac{(m+s-2)!}{m!(s-1)!} (1-p)^{s} \left( p^{m}-(p(1-x))^{m} \right) . \end{aligned}$$

For $s \ne 1$, this gives

58 $$\begin{aligned} E \left[ \frac{1}{A+1} \right]&=\frac{1-p}{(s-1)xp} \left( \sum _{m=1}^{\infty } {m+s-2 \atopwithdelims ()m} (1-p)^{s-1}p^{m} \right. \nonumber \\&\phantom {VV}\left. -\sum _{m=1}^{\infty } {m+s-2 \atopwithdelims ()m} (1-p)^{s-1}(p(1-x))^{m}\right) \end{aligned}$$

59 $$\begin{aligned}&=\frac{1-p}{(s-1)xp} \left( 1-(1-p)^{s-1}+(1-p)^{s-1}-\left( \frac{1-p}{1-p+xp}\right) ^{s-1} \right) \end{aligned}$$

60 $$\begin{aligned}&=\frac{1-p}{(s-1)xp} \left( 1-\left( \frac{1-p}{1-p+px}\right) ^{s-1} \right) . \end{aligned}$$

For $s=1$, the expression from Eq. (57) becomes

61 $$\begin{aligned} {\frac{1-p}{xp} \ln \left( \frac{1-p+xp}{1-p} \right) }. \end{aligned}$$

*FSH:* We have not been able to obtain the key summation term below in closed form, so we simply denote this as $S_{1}$ in Table 3.

62 $$\begin{aligned} S_{1}&=E\left[ \frac{1}{N+1}| A=L-1 \right] P[A=L-1] \end{aligned}$$

63 $$\begin{aligned}&=\sum _{n=L-1}^{\infty } \frac{1}{n+1}\frac{(n+s-1)!}{n!(s-1)!} (1-p)^{s} p^{n} \frac{n!}{(L-1)!(n-L+1)!}x^{L-1}(1-x)^{n-L+1} \end{aligned}$$

64 $$\begin{aligned}&=\frac{ (1-p)^{s}}{(s-1)!(L-1)!}\left( \frac{x}{1-x} \right) ^{L-1} \sum _{n=L-1}^{\infty }\frac{(n+s-1)!}{(n-L+1)!(n+1)} ((1-x)p)^{n}. \end{aligned}$$

*TS:* We have $P[A=L-1]$ from above, so this leaves two more terms. We can give neither of these in closed form, so denote them simply as $S_{2}$ and $S_{3}$, respectively.

65 $$\begin{aligned} S_{2}=P[A<L-1]=\sum _{y=0}^{L-2} {y+s-1 \atopwithdelims ()y} \left( \frac{1-p}{1-p+xp}\right) ^{s} \left( \frac{xp}{1-p+xp}\right) ^{y}, \end{aligned}$$

from Eq. (52). Using Eq. (52) again, we have

66 $$\begin{aligned} S_{3}= & {} \sum _{y=L-1}^{\infty } \frac{1}{y+1} P[A=y]=\sum _{y=L-1}^{\infty } \frac{1}{y+1} {y+s-1 \atopwithdelims ()y}\nonumber \\&\left( \frac{1-p}{1-p+xp}\right) ^{s} \left( \frac{xp}{1-p+xp}\right) ^{y}. \end{aligned}$$

*HD:* By symmetry and following Eq. (60), we have

67 $$\begin{aligned} E \left[ \frac{1}{N-A+1} \right] =\frac{1-p}{(s-1)p(1-x)} \left( 1-\left( \frac{1-p}{1-p+(1-x)p}\right) ^{s-1} \right) . \end{aligned}$$

For $s \ne 1$, we have that

68 $$\begin{aligned} E\left[ \frac{1}{N+1}| A=N \right] P[A=N]&=\sum _{n=0}^{\infty } \frac{1}{n+1}\frac{(n+s-1)!}{n!(s-1)!} (1-p)^{s} p^{n} x^{n}\end{aligned}$$

69 $$\begin{aligned}&=\frac{1-p}{xp(s-1)} \left( \frac{1-p}{1-xp} \right) ^{s-1} \sum _{n=0}^{\infty } \frac{(n+s-1)!}{(n+1)!(s-2)!} (1-xp)^{s-1} (xp)^{n+1}\end{aligned}$$

70 $$\begin{aligned}&=\frac{1-p}{xp(s-1)} \left( \frac{1-p}{1-xp} \right) ^{s-1} \left( 1- (1-xp)^{s-1} \right) . \end{aligned}$$

For $s=1$, Eq. (68) gives

71 $$\begin{aligned} E\left[ \frac{1}{N+1}| A=N \right] P[A=N]=(1-p) \sum _{n=0}^{\infty } \frac{1}{n+1} (xp)^{n}= \frac{1-p}{xp} \ln (1-xp), \end{aligned}$$

and Eq. (67) becomes

72 $$\begin{aligned} E \left[ \frac{1}{N-A+1} \right] =-\frac{1-p}{p(1-x)} \ln \left( \frac{1-p}{1-p+xp}\right) . \end{aligned}$$
